# Scale‐free functional brain dynamics during recovery from sport‐related concussion

**DOI:** 10.1002/hbm.24962

**Published:** 2020-04-29

**Authors:** Nathan W. Churchill, Michael G. Hutchison, Simon J. Graham, Tom A. Schweizer

**Affiliations:** ^1^ Neuroscience Research Program St. Michael's Hospital Toronto Canada; ^2^ Keenan Research Centre for Biomedical Science of St. Michael's Hospital Toronto Canada; ^3^ Faculty of Kinesiology and Physical Education University of Toronto Toronto Canada; ^4^ Physical Sciences Platform Sunnybrook Research Institute Toronto Canada; ^5^ Department of Medical Biophysics University of Toronto Faculty of Medicine Toronto Canada; ^6^ Faculty of Medicine (Neurosurgery) University of Toronto Toronto Canada

**Keywords:** BOLD fMRI, brain injury, concussion, scale free

## Abstract

Studies using blood‐oxygenation‐level‐dependent functional magnetic resonance imaging (BOLD fMRI) have characterized how the resting brain is affected by concussion. The literature to date, however, has largely focused on measuring changes in the spatial organization of functional brain networks. In the present study, changes in the temporal dynamics of BOLD signals are examined throughout concussion recovery using scaling (or fractal) analysis. Imaging data were collected for 228 university‐level athletes, 61 with concussion and 167 athletic controls. Concussed athletes were scanned at the acute phase of injury (1–7 days postinjury), the subacute phase (8–14 days postinjury), medical clearance to return to sport (RTS), 1 month post‐RTS and 1 year post‐RTS. The wavelet leader multifractal approach was used to assess scaling (*c*_1_) and multifractal (*c*_2_) behavior. Significant longitudinal changes were identified for *c*_1_, which was lowest at acute injury, became significantly elevated at RTS, and returned near control levels by 1 year post‐RTS. No longitudinal changes were identified for *c*_2_. Secondary analyses showed that clinical measures of acute symptom severity and time to RTP were related to longitudinal changes in *c*_1_. Athletes with both higher symptoms and prolonged recovery had elevated *c*_1_ values at RTS, while athletes with higher symptoms but rapid recovery had reduced *c*_1_ at acute injury. This study provides the first evidence for long‐term recovery of BOLD scale‐free brain dynamics after a concussion.

## 1 INTRODUCTION

Concussion involves the transmission of impulsive forces to brain tissue, and it is associated with transient impairments in cognition, physical function, and emotion regulation (Daneshvar, Nowinski, McKee, & Cantu, 2011). In the context of sport, the diagnosis and management of concussion are mainly based on symptom assessments, along with brief cognitive and balance testing, with the clinical determination of return to sport (RTS) based on symptom resolution following a graded exercise protocol (McCrory et al., 2013). Although the natural history of clinical recovery is well described, brain recovery following concussion is less understood (McCrea et al., 2017), particularly with respect to time of RTS.

As a mild form of traumatic brain injury (TBI), concussion is rarely associated with gross neuroradiological findings. Instead, injuries at the microscopic level, undetectable in individual patients using standard diagnostic imaging, have a measurable impact on brain function, including disturbances in neurometabolic activity and the regulation of cerebral blood flow (Giza & Hovda, 2014). Blood‐oxygenation‐level dependent functional magnetic resonance imaging (BOLD fMRI) has been used to detect changes in brain function among concussed individuals, during cognitive tasks and at rest (Slobounov, Gay, Johnson, & Zhang, 2012). Studies of the resting brain have typically focused on functional connectivity, which measures the synchrony of spontaneous BOLD signal fluctuations between different brain regions (Sporns, 2014). Functional connectivity has been studied in concussed cohorts, during the symptomatic phase of injury and after RTS (Churchill et al., 2017b; Johnson et al., 2012; Zhang et al., 2012; Zhu et al., 2015), showing significant concussion‐related disturbances that persist beyond medical clearance.

Connectivity‐based methods provide an incomplete picture of the functional brain changes that occur after a concussion, however. These methods do not directly characterize the temporal dynamics of spontaneous BOLD signal fluctuations or provide information about how these dynamics are affected by injury. The temporal dynamics of BOLD signals have been well characterized in the uninjured resting brain and show evidence of scale free (or fractal) behavior, such that no specific timescale plays a dominant role (He, 2014). In the same way that geometric fractals are “self‐similar,” with each magnified part resembling a smaller‐sized copy of the whole (Mandelbrot, 1983), a scale‐free time series signal *x*(*t*) is statistically indistinguishable from a dilated and rescaled version of itself, that is, *x*(*t*)~*a*^−*H*^*x*(*at*), for all values of *a* > 0. In this expression, the Hurst exponent *H* parameterizes scaling behavior, with higher values indicating a more scale‐free signal. In practice, the scaling behavior of functional brain signals is often defined in terms of a power‐law relationship between frequency *f* and the power spectral density (PSD) *P*_*x*_(*f*) ∝ |*f*|^−*β*^, where *β* = 2*H* − 1 (Eke et al., 2000). More recently, sophisticated multifractal formalisms have extended beyond monofractal models in which a single scaling behavior *H* is modeled, instead describing signals in terms of a spectrum of time‐varying scaling exponents *h*, thereby providing a richer description of BOLD dynamics (Ciuciu, Varoquaux, Abry, Sadaghiani, & Kleinschmidt, 2012; Shimizu, Barth, Windischberger, Moser, & Thurner, 2004; Wink, Bullmore, Barnes, Bernard, & Suckling, 2008).

Although it has long been known that the brain exhibits scale‐free dynamics at multiple different levels of organization (Werner, 2010), the initial BOLD fMRI studies of scaling behavior considered it to be a product of measurement noise and/or physiological fluctuations unrelated to brain function (Aguirre, Zarahn, & d'Esposito, 1997; Zarahn, Aguirre, & d'Esposito, 1997). Subsequent studies challenged this assumption though, showing that scale‐free processes in electrophysiology are linked to BOLD signal fluctuations (Van de Ville, Britz, & Michel, 2010). Moreover, BOLD scaling encodes important information, as scaling behaviors vary with cognitive state and systematic differences have been observed in brain areas serving different cognitive roles (Barnes, Bullmore, & Suckling, 2009; Ciuciu et al., 2012; He, 2011). Potentially more relevant to concussion, the suppression of BOLD scaling in the brain is associated with greater cognitive effort (Churchill et al., 2016), trait anxiety (Tolkunov, Rubin, & Mujica‐Parodi, 2010), and distress during adverse life events (Churchill et al., 2015; Tolkunov et al., 2010). Reduced BOLD scaling is also associated with slower reaction time during tasks (Suckling, Wink, Bernard, Barnes, & Bullmore, 2008; Wink et al., 2008). In general, these findings point toward the suppression of BOLD scaling as an indicator of a more taxed, less adaptive brain. This is consistent with the literature on scaling in biological systems (Goldberger et al., 2002), which finds that reduced scaling, which reflects a loss of system complexity, is a hallmark of functional impairment.

Given the literature evidence that suppression of BOLD scaling is a marker of brain dysfunction, this study examined whether concussion, which involves impairments in brain function and behavior including cognitive difficulty and emotional dysregulation, had similar effects on BOLD scaling. It was hypothesized that for concussed athletes: (a) BOLD scaling will show continual increase from acute injury up to 1 month post‐RTS, reflecting brain recovery that lasts beyond medical clearance; (b) for brain areas showing longitudinal change, BOLD scaling will be suppressed relative to uninjured controls at acute injury; and (c) longitudinal recovery will depend on clinical covariates of acute symptom severity and time to RTS. These hypotheses were evaluated using resting‐state BOLD fMRI data acquired from a sample of university athletes with sport‐related concussion who were followed longitudinally from the acute phase of injury to 1 year post‐RTS. In addition, a large normative cohort of athletic controls was also evaluated. Scaling analyses were performed using the wavelet leader multifractal (WLM) formalism, which offers superior performance over standard monofractal techniques (Lashermes, Jaffard, & Abry, 2005; Wendt & Abry, 2007; Wendt, Abry, & Jaffard, 2007).

## 2 METHODS

### 2.1 Study participants

Sixty‐one concussed athletes were recruited from university‐level sport teams at a single institution (including volleyball, hockey, soccer, football, rugby, basketball, lacrosse, and water polo; see Supplementary Table S1 for athlete numbers by sport) through the academic sport medicine clinic, following a concussion diagnosis. Diagnosis was determined by a staff physician following a sustained direct or indirect contact to the head with signs and/or symptoms as per the Concussion in Sport Group guidelines (McCrory et al., 2013). Imaging evaluations were conducted at the acute phase of injury (ACU; 1–7 days postinjury), the subacute phase (SUB; 8–14 days postinjury), medical clearance to RTS (RTS), 1 month post‐RTS (1MO) and 1 year post‐RTS (1YR). Within the longitudinal study, some of the concussed athletes had missed imaging sessions. The number of participants retained at each time point was: ACU (53/61), SUB (29/61), RTS (51/61), 1MO (45/61), and 1YR (32/61). Attrition was not significantly related to demographic variables (age, sex, concussion history) or clinical variables (symptom severity, time to RTS) examined in this study, based on Spearman correlations, at a false discovery rate (FDR) of 0.05 across time points.

As a control group, one hundred and sixty‐seven athletes were also consecutively recruited and imaged at the start of their competitive season. All athletes in the study completed baseline assessments with the Sport Concussion Assessment Tool (SCAT) (Echemendia et al., 2017; Guskiewicz et al., 2013) before the beginning of their seasons. Athletes diagnosed with a concussion also completed SCAT assessments at acute injury and at time of RTS. Athlete recruitment and data collection were carried out between October 2014 and March 2019. None of the athletes in this study had a history of neurological or psychiatric diseases or sensory/motor impairments, and none of the concussed athletes experienced loss of consciousness or posttraumatic amnesia. This study was carried out in accordance with recommendations of the Canadian Tri‐Council Policy Statement 2 and with approval of the research ethics boards at the University of Toronto and St. Michael's Hospital, with written informed consent given by all participants in accordance with the Declaration of Helsinki.

### 2.2 Magnetic resonance imaging

The athletes were all imaged at St. Michael's Hospital using a research‐dedicated MRI system operating at 3 Tesla (Magnetom Skyra, Siemens, Erlangen, Germany) with the standard 20‐channel head receiver coil. Structural imaging included three‐dimensional T1‐weighted magnetization prepared rapid acquisition gradient echo (3D MPRAGE: inversion time (TI)/echo time (TE)/repetition time (TR) = 1,090/3.55/2,300 ms, flip angle (FA) = 8°, 192 sagittal slices with field of view (FOV) = 240 × 240 mm^2^, 256 × 256 pixel matrix, 0.9 mm slice thickness, 0.9 × 0.9 mm in‐plane resolution, with bandwidth (BW) = 200 Hz per pixel (Hz/px)), fluid attenuated inversion recovery imaging (FLAIR: TI/TE/TR = 1,800/387/5,000 ms, 160 sagittal slices with FOV = 230 × 230 mm^2^, 512 × 512 matrix, 0.9 mm slice thickness, 0.4 × 0.4 mm^2^ in‐plane resolution, BW = 751 Hz/px) and susceptibility‐weighted imaging (SWI: TE/TR = 20/28 ms, FA = 15°, 112 axial slices with FOV = 193 × 220 mm^2^, 336 × 384 matrix, 1.2 mm slice thickness, 0.6 × 0.6 mm^2^ in‐plane resolution, BW = 120 Hz/px). Structural images were reviewed in a two‐step procedure, involving initial inspection by an MRI technologist during the imaging session and later review by a neuroradiologist with clinical reporting, if abnormalities were identified. Statistical testing was also performed by obtaining the mean, variance and skew of voxel signal intensity distributions for masked MPRAGE, FLAIR, and SWI images (following brain extraction using FSL *bet* software), generating a Z‐score for each imaging sequence per athlete relative to the controls and identifying significant outliers at *p* < .05. No abnormalities (white matter [WM] hyperintensities, contusions, microhemorrhage, or statistical outliers) were found for study participants.

Resting‐state fMRI data were acquired via multislice T2*‐weighted echo planar imaging (TE/TR = 30/2,000 ms, FA = 70°, 32 oblique‐axial slices with FOV = 200 × 200 mm^2^, 64 × 64 matrix, 4.0 mm slice thickness with 0.5 mm gap, 3.125 × 3.125 mm^2^ in‐plane resolution, BW = 2,298 Hz/px), producing a time series of 195 images at each slice location. During fMRI, athletes were instructed to lie still with their eyes closed and not to focus on anything in particular. Processing and analysis were performed using the Analysis of Functional Neuroimages (AFNI) package (afni.nimh.nih.gov), fMRIB Software Library (FSL; https://fsl.fmrib.ox.ac.uk), and customized algorithms developed in the laboratory. After discarding the first four volumes to allow the fMRI signal to reach equilibrium, the processing included rigid‐body motion correction (AFNI *3dvolreg*), removal of outlier scan volumes using the SPIKECOR algorithm (nitrc.org/projects/spikecor), slice‐timing correction (AFNI *3dTshift*), spatial smoothing with a 6 mm full width at half maximum (FWHM) isotropic 3D Gaussian kernel (AFNI *3dmerge*) and regression of motion parameters and linear‐quadratic trends as nuisance covariates. For motion parameter regression, principal component analysis was performed on the six rigid‐body movement parameters (consistently accounting for >85% of variance), and the first two PCs were used as nuisance regressors. To control for physiological noise, the data‐driven PHYCAA+ algorithm (nitrc.org/projects/phycaa_plus) was used to downweight areas with nonneural signal, followed by further regression of signal originating from WM and cerebrospinal fluid (CSF). The WM and CSF regressions were performed after spatial normalization, described in the paragraph below.

To perform group‐level analyses, the fMRI data were coregistered to a common anatomical template using the FMRIB Software Library (FSL) package (https://fsl.fmrib.ox.ac.uk). The FSL *flirt* algorithm was used to compute the rigid‐body transform of the mean functional volume for each athlete to their T1‐weighted anatomical image, along with the 12‐parameter affine transformation of the T1 image for each athlete to the MNI152 template. The net transform was applied to the functional imaging data, which was resampled at 3 × 3 × 3 mm^3^ resolution. To remove WM and CSF signal, subject T1‐weighted images were segmented and coregistered to the MNI152 template using the *fslvbm* protocol (fsl.fmrib.ox.ac.uk/fsl/fslwiki/FSLVBM), which used *fast* to obtain partial volume segmentation maps of gray matter (GM), WM, and CSF, followed by iterative applications of affine registration algorithm *flirt* and nonlinear registration algorithm *fnirt*, to obtain a symmetric, study‐specific mean GM tissue template. The spatial transforms were subsequently used to obtain mean WM and CSF tissue templates, resampled into 3 × 3 × 3 mm^3^ resolution and a 6 mm FWHM isotropic 3D Gaussian smoothing kernel was applied. For WM, the brain mask *p*(WM) ≥ *P*_95%_(WM) was obtained (i.e., voxels within the distribution 95th percentile) and a single spatial erosion performed (3 × 3 kernel, in‐plane). Two mean seed time series were obtained by separately averaging over cerebral WM voxels and averaging over brainstem WM (as their time courses were substantially different). For CSF, the brain mask *p*(CSF) ≥ *P*_95%_(CSF) was obtained and manually edited into two separate masks of the lateral ventricles. Two mean seed time series were obtained by separately averaging over these two ventricular regions. The four physiological time series were then regressed from each voxel, for all study participants.

To reduce computational burden and improve the stability of regional BOLD measures, the data were then parcellated using the Brainnetome Atlas (BNA), which a connectivity‐based atlas that subdivides the brain into 210 cortical regions and 36 subcortical regions (Fan et al., 2016). Within each parcel, a mean seed time series of interest was obtained, for a cubic region of interest (ROI) of 3 × 3 × 3 voxels, placed at the parcel center of mass. Seed ROIs of uniform size were created for all parcels, to avoid potential unequal smoothing effects across caused by averaging over parcels of different size. This procedure generated a set of 246 BOLD time series per participant for subsequent analyses.

### 2.3 Clinical and demographic data

Participant demographics are reported in Table 1, including age, sex, and prior concussion history, along with time to RTS for concussed athletes, defined as the number of days from concussion event to symptom resolution following a graded exertional protocol (McCrory et al., 2017). From the SCAT, a symptom severity score was obtained by summing across the 22‐item symptom scale, with each item receiving a 7‐point Likert scale rating. A total symptoms score was also obtained by counting the total number of symptoms with nonzero ratings. In addition, brief cognitive testing scores were reported, including orientation, immediate memory, concentration, and delayed memory, along with total scores for the modified balance error scoring system. All scores were tested at acute injury and RTS for a significant difference relative to baseline, via nonparametric Wilcoxon paired‐measures tests (two‐tailed). As per evolving clinical guidelines, the first 44/61 concussed athletes and 68/167 controls in this study were evaluated with SCAT3, while the remainder were evaluated using SCAT5. The immediate memory and delayed memory tests were changed in SCAT5 (from 15 to 30 items and from 5 to 10 items, respectively). For these subtests, statistics are only reported for SCAT3 data, as this represents the larger sample of concussed athletes. For all other subtests, statistics are based on the complete dataset, after verifying that there were no significant within‐cohort differences in SCAT3 and SCAT5 scores based on two‐sample Wilcoxon tests (*p* ≥ .288, for all measures).

**Table 1:** Demographic and clinical data for athletes with concussion and controls. Clinical scores of total symptoms and symptom severity are summarized by the median [Q1, Q3]. For tests of Immediate Memory and Delayed Memory, denoted by a “*,” statistics are based on a reduced sample of 44/61 concussed athletes and 68/167 controls, due to changes in scoring guidelines between SCAT3 and SCAT5. Significant differences in scores at acute injury, relative to baseline, are noted with “**”

	Control		Concussion	
Age (mean ± *SD*)	20.2 ± 2.0		20.4 ± 2.0	
Female	87/167 (52%)		31/61 (51%)	
Previous concussions	73/167 (44%)		36/61 (59%)	
Days to RTP	—		30 [15, 66]	

		Baseline	ACU	RTS
Total symptoms	2 [0, 5]	3 [1, 5]	10 [5, 17]**	1 [0, 2]
Symptom severity	3 [0, 7]	3 [1, 10]	14 [6, 35]**	1 [0, 2]
Orientation	5 [5, 5]	5 [5, 5]	5 [5, 5]	5 [5, 5]
Immediate memory*	15 [15,15]	15 [14, 15]	15 [14, 15]	15 [15, 15]
Concentration	4 [3, 5]	4 [3, 5]	4 [3, 5]	4 [3, 5]
Delayed memory*	4 [3, 5]	5 [4, 5]	4 [3, 5]	5 [4, 5]
M‐BESS errors	2 [1, 4]	3 [2, 5]	4 [1, 6]	1 [0, 5]

Abbreviations: M‐BESS, modified balance error scoring system; RTS, return to sport.

### 2.4 Measuring BOLD scaling

The goal of the analyses in this section was to summarize the dynamics of the spontaneous, arrhythmic fluctuations of resting‐state BOLD time series *x*(*t*). This was attained for each of the 246 parcel ROIs identified for a given participant (concussed and control) and imaging session. One approach involves analyzing signal variance at different frequencies *f* using a PSD estimator. However, both the BOLD signal and underlying neural activity have a broad spectral distribution, where power declines smoothly with increasing frequency (Fox, Snyder, Vincent, & Raichle, 2007); hence, no specific set of frequencies or timescales can be singled out for analysis. For this reason, *scaling analysis* is an appealing alternative; instead of analyzing specific frequencies, this approach characterizes power‐law scaling behaviors that relate the dynamics of BOLD signals at different timescales. By convention, *x*(*t*) is deemed *scale invariant* if the following relation holds for a wide range of frequencies:

(1) $$P_{x}\left(f\right)=C{\left|f\right|}^{-\beta }\;\left(\beta >0\right)$$

This has nontrivial implications, as the relative spectral power at a given frequency *f* is then entirely dictated by a single scaling exponent *β*. This parameter is also related to the Hurst exponent by *β* = 2*H* − 1 for a fractional Brownian motion model (Eke et al., 2000), where *H* is considered an index of temporal dependence. A value of 0 < *H* < 0.5 indicates short‐range dependency, where a high value for *x*(*t*) tends to be followed by a low value for *x*(*t* + 1) and vice‐versa; *H* = 0.5 denotes an uncorrelated process; and 0.5 < *H* < 1.0 indicates long‐range dependency, where a high (or low) value for *x*(*t*) tends to be followed by a high (or low) value for *x*(*t* + 1). In practise, PSD‐based scaling analysis typically involves estimating *β* from the slope of log(*P*_*x*_(*f*)) versus log(*f*) scatterplots, calculated using standard linear regression techniques.

Although intuitive, PSD‐based scaling analyses suffer from a significant limitation, namely the inability to distinguish true scaling behavior from deterministic trends in the data. To address this issue, scaling may be evaluated using wavelets (Abry, Gonçalvés, & Flandrin, 1995; Abry & Veitch, 1998; Veitch & Abry, 1999). The wavelet transform employs translated and dilated versions of a basis function Ψ_0_(*t*), which is compact in both time and frequency domains, to analyze *x*(*t*) at different delays and timescales. This basis function Ψ_0_(*t*) is also characterized as having *N*_Ψ_ ≥ 1 vanishing moments, where for all integers *k* = 0, …, *N*_Ψ_ − 1, ∫*t*^*k*^Ψ_0_(*t*)*dt* = 0, making Ψ_0_(*t*) insensitive to low‐order polynomial trends in *x*(*t*) (Daubechies, 1992). In the continuous time domain, the wavelet coefficient *d*_*x*_(*a*, *k*) measures signal at timescale *a* and delay *k* by computing the inner product $d_{x}\left(a,k\right)=\frac{1}{a}\int x\left(t\right)\Psi_{0}\left(\frac{t-k}{a}\right)\mathrm{dt}$. In discretely sampled real‐world data, the analogous computation uses the discrete wavelet transform (DWT), which analyzes dyadic scales *a* = 2^*j*^ and delay intervals *k* = *n*2^*j*^, for nonnegative integer *j* and *n*. The mean squared wavelet energy at a given timescale is then calculated as ${\left|d_{x}\left(2^{j}\right)\right|}^{2}=\frac{1}{N}\sum_{n}{\left|d_{x}\left(2^{j},n2^{j}\right)\right|}^{2}$. The wavelet energy has a relationship with dyadic scale that is described by the following equation:

(2) $${\left|d_{x}\left(2^{j}\right)\right|}^{2}=C2^{\mathrm{j\beta}}\;$$

The DWT approach shows reduced modeling error compared to PSD estimators (Abry et al., 1995; Abry & Veitch, 1998) and is thus the preferred technique for scaling analyses. As with PSD‐based methods, scaling analysis typically involves estimating *β* from the slope of log(|*d*_*x*_(2^*j*^)|^2^) versus *j* scatterplots.

One additional concern is that the DWT approach assumes the data of interest to be Gaussian, with scaling of *x*(*t*) fully defined by its variance (i.e., its second‐order moment) across timescales. We may extend the approach to encompass scaling behaviors for a range of moments *q* using the WLM formalism (Lashermes et al., 2005; Wendt & Abry, 2007; Wendt et al., 2007). To ensure robust performance, wavelet coefficients *d*_*x*_(*a*, *k*) are replaced with wavelet leaders *L*_*x*_(*a*, *k*), which are calculated as the largest coefficient value |*d*_*x*_(*a*′, *k*′)| in a neighborhood of *k*, for all *a* ′  ≤ *a*. The WLM approach then describes a more general expression relating dyadic timescale *j* to wavelet power:

(3) $$\frac{1}{K}\sum_{k}{\left|L_{x}\left(2^{j},k\right)\right|}^{q}=C_{q}2^{\mathrm{j\tau}\left(q\right)}\;$$

Instead of a fixed *β* value, wavelet power scaling is now expressed in terms of a characteristic function *τ*(*q*) that varies with statistical moment *q*. This function is typically expressed as a Legendre polynomial expansion *τ*(*q*) ≈ ∑_*p*_*c*_*p*_(*q*^*p*^/*p*!), where the set of log‐cumulants *c*_*p*_ summarize scaling behavior of *x*(*t*).

The function *τ*(*q*) is linked to the concept of multifractality, in which *x*(*t*) exhibits time‐varying fractal features characterized by their Hölder exponent values *h*(*t*), which describe power‐law scaling behavior of *x*(*t*) in the neighborhood of each time point *t* (Wendt & Abry, 2007). This collection of scaling exponents is characterized by a “singularity spectrum” *D*(*h*), which measures the Hausdorff dimension at each value *h*, which is the “size” of the set of time points *t*_*i*_ such that *h*(*t*_*i*_) = *h*. In practise, *D*(*h*) is approximated using the Legendre transform of *τ*(*q*) and therefore the log‐cumulants *c*_*p*_ reflect multifractal scaling behavior.

The present work examined the parameterization *τ*(*q*) = *c*_1_*q* + *c*_2_*q*^2^/2, as higher order coefficients were found to be nonsignificant under bootstrap testing (Wendt & Abry, 2007). The log‐cumulant *c*_1_ is equivalent to the peak amplitude of *D*(*h*), that is, the most frequently‐occurring scaling parameter *h*, which is similar to monofractal *H* (Wendt et al., 2007). The log‐cumulant *c*_2_ determines the width of *D*(*h*), and can therefore be thought of as indexing the degree of multifractality (Ciuciu et al., 2012). Multifractal analysis was performed using the WLBMF toolbox (https://www.irit.fr/~Herwig.Wendt/software.html; [Wendt & Abry, 2007; Wendt et al., 2007]). The WLM estimation was performed using Daubechies' wavelets with *N* = 3 vanishing moments and *q* values ranging −10 to 10. Robust results were identified for dyadic wavelets over a three octaves range of *j* = [2, 4], which corresponds to a frequency range of [0.016, 0.125] Hz.

### 2.5 Analysis of healthy controls

To characterize the spatial distribution of scaling behavior, the *c*_1_ and *c*_2_ log‐cumulant values were obtained for all athletic controls, at each of the 246 ROIs. Bootstrap 1‐sample tests were then conducted at each ROI to evaluate scaling behavior: *c*_1_ was tested against the null *c*_1_ ≤ 0.5 (1‐tailed) and *c*_2_ was tested against the null *c*_2_ = 0 (2‐tailed). Significant ROIs were then identified after adjusting for multiple comparisons at an FDR of 0.05. For ROIs that were determined to be significant, maps of the group mean and standard error values were displayed.

Subsequent analyses examined whether both *c*_1_ and *c*_2_ were affected by demographic factors. At each ROI, log‐cumulant values were regressed onto covariates of age (integer), sex (binary) and history of concussion (binary) using a general linear model (GLM). To minimize assumptions about the distribution of log‐cumulant values, bootstrapping was used to obtain empirical *p*‐values on the regression coefficient values *b*. The ROIs showing a significant effect of imaging session were then identified at an FDR threshold of 0.05. Afterwards, log‐cumulant values were averaged across significant ROIs and the values reanalyzed with a bootstrapped GLM to obtain a single set of summary statistics, including coefficient *b* with a 95% confidence interval (95% CI), standardized measure of effect size termed the bootstrap ratio (BSR; calculated as *b* divided by bootstrapped standard error) and *p*‐value.

### 2.6 Longitudinal effects of concussion

To model longitudinal change in *c*_1_ and *c*_2_ values of concussed athletes (see Section 2.1), the effect of imaging session on log‐cumulant values was evaluated at each ROI using a linear mixed‐effects model (LMM). For this model, missing longitudinal data are handled implicitly via maximum likelihood estimation. Fixed effects were estimated at each post‐acute imaging sessions (SUB, RTS, 1MO, 1YR) relative to ACU; the model also included fixed‐effect covariates adjusting for age, sex, and concussion history and subject‐specific random‐effects intercepts. For seven participants who were medically cleared at the time of ACU or SUB scan, the corresponding datapoints were given labels of both ACU/SUB and RTS during model fitting. The LMMs were fitted using the MATLAB R2017b *fitlme* package (The MathWorks, Natick, MA) with full covariance estimation using Cholesky parameterization. Analysis was done in a bootstrap resampling framework, where resampling units consisted of the set of all images for a given subject (1,000 iterations). This was used to obtain empirical *p*‐values on the fixed‐effect regression coefficients *b*. The ROIs showing a significant effect of imaging session were then identified at an FDR threshold of 0.05. Afterwards, log‐cumulant values were averaged across significant ROIs and the values reanalyzed with a bootstrapped LMM to obtain summary statistics, including fixed‐effect coefficient *b* with a 95%CI, BSR and *p*‐value for each imaging session.

For ROIs showing significant longitudinal change, the *c*_1_ and *c*_2_ values of concussed athletes were also compared to athletic controls. Log‐cumulant values were averaged over all ROIs showing significant longitudinal effects, and at each imaging session (ACU, SUB, RTS, 1MO, 1YR) concussed and control group means were compared. Given the demographic effects seen in the control cohort (see Section 2.5), a GLM was used to evaluate the effects of concussion with covariates adjusting for age, sex and history of concussion. This was done in a bootstrap resampling framework (1,000 iterations) to obtain coefficient *b* with a 95%CI, BSR, and *p*‐value for each imaging session, with significant imaging sessions identified at an FDR threshold of 0.05. To mitigate potential bias and loss of efficiency due to missing data, bootstrap GLM analysis was combined with multiple imputation using the “Boot MI” approach of (Schomaker & Heumann, 2018): bootstrap samples were drawn from the full dataset (including missing data) and for each sample, imputation was done *M* = 10 times to generate 10 coefficient estimates, which were averaged to obtain a point estimate. The set of coefficient point estimates were treated as a conventional bootstrap empirical distribution, from which summary statistics were calculated. Imputation was done using the fitted LMM to generate simulated log‐cumulant values. To evaluate the impact of imputation, results of MI analyses were also compared to unimputed bootstrap parameter estimates in Table S1.

### 2.7 Effects of clinical covariates

A secondary set of analyses, performed within the concussed cohort, tested for effects of clinical covariates on log‐cumulant values over the course of recovery. This included (a) total symptom severity at acute injury and (b) days to RTS. Given the high correlation between these variables (see Section 3.1), a pair of orthogonal composite scores (CS) were defined. After the two variables were renormalized via the inverse empirical distribution function and mean centered, composite score one (CS1) was computed as the average of the two variables (symptoms + days to RTP), which quantified overall clinical outcome. Composite score two (CS2) was computed as the difference (symptoms − days to RTP), which quantified discrepancy between the two measures of concussion outcome (i.e., a positive score denoting high symptom burden but rapid recovery, and a negative score denoting the converse).

The effects of these covariates on log‐cumulant values were assessed within the previously established bootstrapped LMM framework. In addition to fixed‐effect covariates of imaging session (SUB, RTS, 1MO, 1YR) and demographics (age, sex, concussion history), the model was augmented by adding interaction effects of CS1 and CS2 at each imaging session (i.e., measuring the simple effects on concussed athletes). Bootstrap resampling was done to obtain empirical *p*‐values on the fixed‐effect regression coefficients *b*. The ROIs showing a significant effect of imaging session were then identified at an FDR threshold of 0.05. Afterwards, log‐cumulant values were averaged across significant ROIs and the values reanalyzed with a bootstrapped LMM to obtain summary statistics, including a fixed‐effect coefficient *b* with a 95%CI, BSR, and *p*‐value.

## 3 RESULTS

### 3.1 Demographic and clinical data

The demographic and clinical data are summarized in Table 1. Both concussed and control groups were of similar age, and included similar proportions of male and female athletes, with and without prior history of concussion. At baseline, concussed athletes had symptom scores comparable to controls. At ACU, both total symptoms and symptom severity were significantly elevated relative to their baseline values (*p* < .001 for both), whereas at RTS they were significantly lower than baseline (*p* < .001 for both), with all effects significant at an FDR of 0.05. For other cognitive and balance tests, no significant effects were identified at acute injury or at RTS (*p* ≥ .31 for all tests). Median time to RTS, which included symptom resolution and completion of a graded exercise protocol, was approximately 1 month, with an interquartile range of 2 weeks to 2 months. These times tended to be slightly long compared to consensus guidelines (McCrory et al., 2017), but not unreasonable given the young age and heterogeneous mixture of male and female athletes drawn from contact and noncontact sports. The symptom severity scores at early injury had a relatively high Spearman correlation with days to RTS of 0.561 (95%CI = 0.379–0.696; *p* < .001), indicating that individuals with greater initial symptoms also tended to have a more prolonged recovery.

### 3.2 Scaling behavior in healthy controls

**Figure 1:**
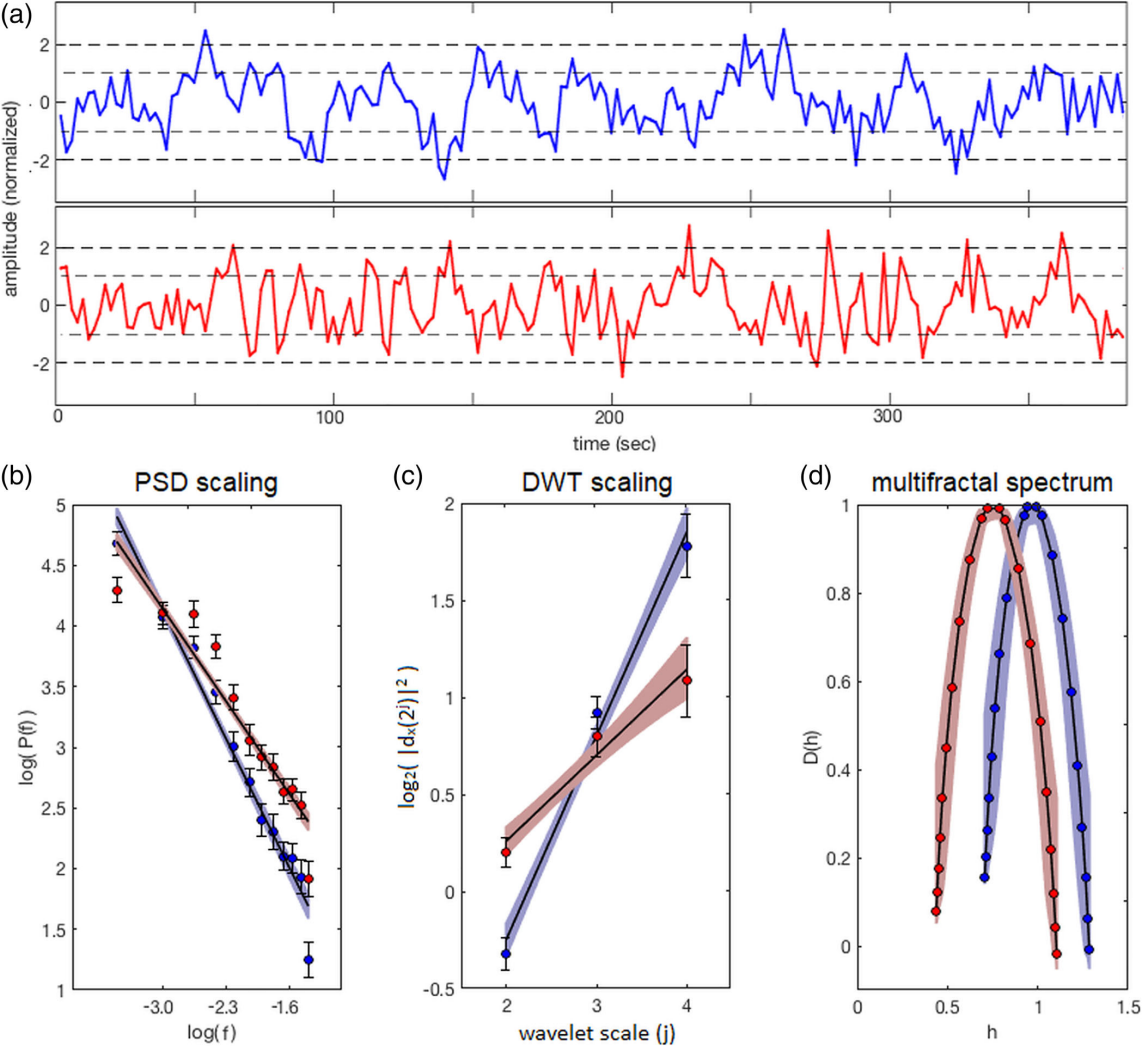
Illustration of BOLD scaling behavior, comparing example ROIs in the right precuneus (blue) and right putamen (red) for healthy control athletes. (a) Sample BOLD time series data from a representative participant. (b) Log–log plot of spectral power versus frequency used to estimate PSD scaling. (c) Log‐linear plot of wavelet power versus timescale for DWT estimation. (d) Plot of estimated Hausdorff dimensionality *D*(*h*) versus Hölder exponent *h*, obtained using WLM techniques. The precuneus ROI is located in right medial area 7 (PEp); CoM = 4, −66, 50, and the basal ganglia ROI is located in the right dorsolateral putamen; CoM = 30, −2, 4. BOLD, blood‐oxygenation‐level‐dependent; DWT, discrete wavelet transform; ROI, region of interest; WLM, wavelet leader multifractal

Figure 1 illustrates BOLD scaling behavior for representative time series data obtained from ROIs in the right precuneus (medial area 7 [PEp], CoM = −4, −66, 50) and right basal ganglia (dorsolateral putamen, CoM = 30, −2, 4) of healthy control athletes. Figure 1a shows sample BOLD time series of a single athlete, where the precuneus exhibits more complex behavior, with faster small fluctuations superimposed on slower, smooth signal variations. Conversely, the putamen exhibits more random fluctuations, better approximating white noise. This difference in dynamics is expressed in the log‐scale plots of Figure 1b–d, obtained by averaging over all control athletes. When comparing the precuneus relative to the putamen, a steeper negative slope is evident in the plot of log(*P*_*x*_(*f*)) versus log(*f*) and a steeper positive slope is evident in the plot of log_2_(*d*_*x*_(2^*j*^)) versus *j* used for DWT estimation. Similarly, the plots of multifractal spectrum *D*(*h*) versus *h* obtained using WLM techniques show that the spectrum peak (i.e., *c*_1_) of the precuneus is shifted to the right, denoting increased scaling behavior relative to the putamen. In addition, these spectra show relatively broad curves, denoting the presence of multifractal scaling in both regions (i.e., nonzero *c*_2_).

**Figure 2:**
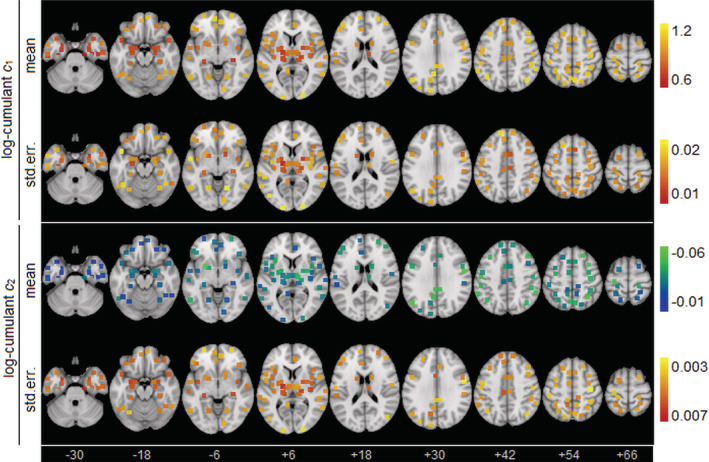
Depiction of scaling coefficient values *c*_1_ and *c*_2_ for healthy control athletes, including group mean and standard error of the mean (*SE*). Regions of interest (ROIs) are shown as square patches and the MNI z‐axis coordinate is listed below each slice

One‐sample tests of the log‐cumulants established significance for both scaling parameter *c*_1_ and multifractal parameter *c*_2_ for all brain ROIs, at an FDR of 0.05. For *c*_1_, the global average value computed over all ROIs was near unity (median, [Q1, Q3]: 0.911, [0.843, 0.969]), denoting highly scale‐free resting‐state BOLD fMRI signals. For *c*_2_, the global average value computed over all ROIs was small in magnitude but consistently negative (−0.037 [−0.049, −0.026]), denoting significant multifractality with a concave characteristic function *τ*(*q*), as expected. Figure 2 displays regional group means and standard errors of the log‐cumulants, for the control athlete cohort. The highest *c*_1_ and lowest *c*_2_ values were predominantly in frontal, parietal and cingulate regions. Conversely, lower *c*_1_ and higher *c*_2_ values are generally observed subcortically, along with the temporal poles. In general, there was modest but significant spatial correlation between the mean *c*_1_ and *c*_2_ maps, with a Spearman correlation of −0.431 (95%CI: −0.484, −0.274), indicating that brain regions with greater fMRI signal scaling behavior also tended to exhibit greater multifractality.

The GLM analyses of controls revealed significant effects of demographics on *c*_1_, with ROIs summarized in Table 2, whereas no significant effects on *c*_2_ were identified. Among control athletes, greater age was significantly associated with increased *c*_1_ in somatosensory and motor areas. Averaging over significant brain regions, a *b* coefficient value of 0.031 was obtained (95%CI: 0.020, 0.041; BSR = 6.18; *p* < .001). Sex was associated with more spatially extensive differences, as female athletes had lower *c*_1_ values compared to male athletes throughout the brain, including prefrontal, occipital, and subcortical regions. Averaging over significant regions, a *b* value of −0.111 was obtained (95%CI: −0.142, −0.077; BSR = −6.73; *p* < .001). No significant effects of history of concussion on *c*_1_ were identified.

**Table 2:** Summary of ROIs identified in control athletes that show significant effects on *c*_1_ for demographic factors of age, sex or history of concussion (conc.hx.). Standardized effect sizes are reported in terms of BSRs. Significant effects are noted with “*” (FDR = 0.05 threshold). Brain regions are defined based on the BNA

	Brain region	Center of mass (MNI coordinates)			BSR		
					Age	Sex	conc.hx.
1	Middle frontal gyrus L (A9/46d)	−27	42	30	−1.05	−4.81*	1.10
2	Orbital gyrus L (A14m)	−6	54	−9	−0.11	−3.21*	1.48
3	Precentral gyrus R (A4ul)	36	−18	57	3.56*	−1.22	0.09
4	Precentral gyrus R (A4t)	15	−21	72	3.95*	0.14	1.39
5	Paracentral lobule R (A4ll)	6	−21	60	3.79*	−2.01	0.16
6	Fusiform gyrus R (A20rv)	33	−15	−30	−0.54	−4.77*	1.24
7	Postcentral gyrus L (A1/2/3tru)	−21	−33	69	3.65*	−0.63	0.53
8	Medioventral occipital cortex L (cLinG)	−12	−81	−12	0.80	−3.32*	0.23
9	Lateral occipital cortex L (iOccG)	−30	−87	−12	−0.24	−3.34*	1.89
10	Basal ganglia L (vmPu)	−24	6	−3	1.04	−3.48*	1.75
11	Basal ganglia R (dlPu)	30	−3	3	0.71	−3.59*	0.41

*Note*: A9/46d = dorsal area 9/46, A14m = medial area 14, A4ul = area 4 (upper limb region), A4t = area 4 (trunk region), A4ll = area 4 (lower limb region), A20rv = rostroventral area 4, A1/2/3tru = area 1/2/3 (trunk region).

Abbreviations: BNA, Brainnetome Atlas; BSR, bootstrap ratio; cLinG, caudal lingual gyrus, dlPu, dorsolateral putamen; FDR, false discovery rate; iOccG, inferior occipital gyrus; vmPu, ventromedial putamen.

### 3.3 Main effects of concussion

**Figure 3:**
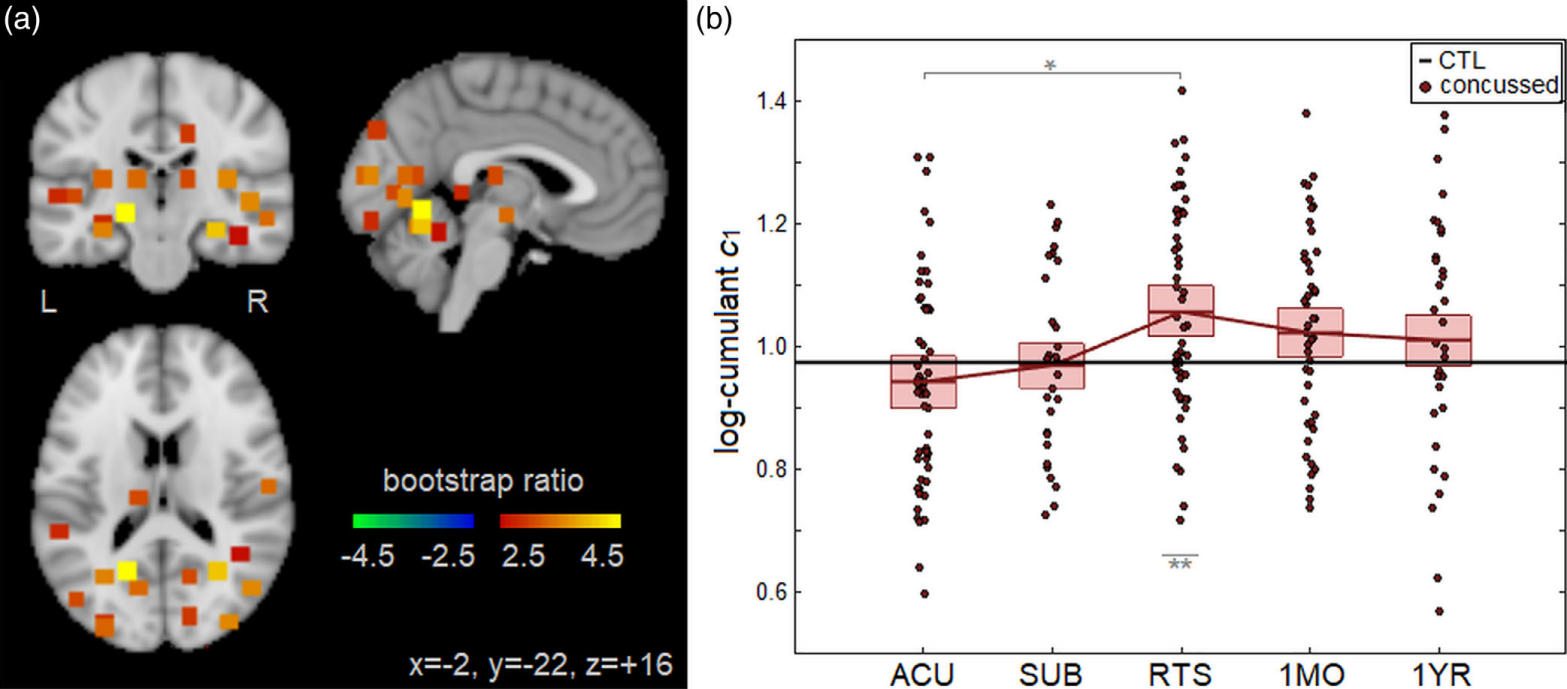
(a) Brain regions of interest (ROIs; square patches) showing significant longitudinal change in *c*_1_ over the course of concussion recovery. (b) Distribution of concussed athlete *c*_1_ values, averaged over significant ROIs, plotted for each imaging session. For the distribution plot, horizontal red lines denote group means and boxes indicate 95% confidence intervals; distribution means are connected between sessions by solid red lines; the mean *c*_1_ value for controls is plotted as the thick horizontal black line. The *c*_1_ values were obtained from ROIs showing significant longitudinal change from ACU to RTP, noted with “*” and significant cross‐sectional difference relative to controls is noted with “**” (false discovery rate [FDR] = 0.05 threshold)

The LMM analyses of concussed athletes showed significant effects of imaging session on *c*_1_, with ROIs summarized in Table 3, whereas no significant effects on *c*_2_ were identified. Significant changes in *c*_1_ relative to ACU were predominantly seen at RTS, with a single ROI (left medioventral occipital cortex), remaining significant at 1MO and two additional ROIs (left superior frontal and inferior frontal gyri) emerging as significant at this time; no significant effects were identified at 1YR. Figure 3a depicts the brain areas of significant longitudinal change. The ROIs were predominantly within occipital brain areas, with a smaller set in temporal and thalamic areas. All significant ROIs had uniformly positive BSRs, denoting a longitudinal increase in *c*_1_ from ACU to RTS. Figure 3b plots the distribution of participant *c*_1_ values as a function of imaging session, averaged over significant ROIs. The *c*_1_ values had increased from ACU to RTS, followed by a decline until 1YR. Table 4 reports the LMM summary statistics for *c*_1_ values averaged over significant brain regions, showing that the greatest change relative to ACU was at RTS, in both absolute and standardized differences. Examining demographic covariates within the significant ROIs, age did not show significant effects on *c*_1_ after concussion (*b* = 0.001; 95%CI: −0.011, 0.013; BSR = 0.02; *p* = .962), but female athletes had lower values (*b* = −0.105; 95%CI: −0.152, −0.061; BSR = ‐4.56; *p* < .001), as did athletes with a history of concussion (*b* = −0.060; 95%CI: −0.109, −0.007; BSR = ‐2.28; *p* = .028). Table 4 also reports the two‐sample analysis results comparing concussed athletes to controls. The *c*_1_ values for concussed athlete were lower than controls at ACU, but the effects were nonsignificant. However, the *c*_1_ values for concussed athletes were significantly elevated relative to controls at RTS, before becoming nonsignificant at later imaging sessions.

**Table 3:** Summary of ROIs identified in concussed athletes that show significant longitudinal change in *c*_1_ relative to ACU, as displayed in Figure 4. Standardized effect sizes are reported in terms of BSRs. Significant effects are noted with “*” (FDR = 0.05 threshold). Brain regions are defined based on the BNA

	Brain region	Center of mass			BSR			
		(MNI coordinates)			SUB	RTS	1MO	1YR
1	Superior frontal gyrus L (A10m)	−9	57	15	0.97	0.85	3.10*	0.06
2	Inferior frontal gyrus L (A45r)	−48	36	−3	−0.16	0.64	3.15*	0.70
3	Middle temporal gyrus R (ASTS)	57	−15	−9	1.69	3.33*	1.90	1.50
4	Fusiform gyrus L (A37mv)	−30	−63	−15	−0.08	3.52*	1.48	1.28
5	Fusiform gyrus R (A37mv)	30	−60	−15	0.62	4.06*	1.54	1.16
6	Fusiform gyrus R (A37lv)	42	−51	−18	1.14	2.60*	1.72	1.55
7	Posterior superior temporal sulcus L (rpSTS)	−54	−39	3	0.23	2.81*	2.72	1.25
8	Medioventral occipital cortex L (rLingG)	−18	−60	−6	0.39	5.18*	3.70*	0.78
9	Medioventral occipital cortex L (vmPOS)	−12	−69	12	0.72	3.32*	2.78	0.74
10	Medioventral occipital cortex R (vmPOS)	15	−63	12	0.51	3.07*	2.22	1.05
11	Lateral occipital cortex L (mOccG)	−30	−90	12	1.42	3.29*	1.34	2.19
12	Lateral occipital cortex R (mOccG)	36	−87	12	0.54	3.56*	1.65	1.76
13	Lateral occipital cortex L (V5/MT+)	−45	−75	3	1.44	3.07*	1.97	1.36
14	Lateral occipital cortex R (V5/MT+)	48	−69	0	2.27	3.57*	2.73	2.59
15	Lateral occipital cortex L (iOccG)	−30	−87	−12	1.11	2.83*	0.73	1.23
16	Lateral occipital cortex R (msOccG)	15	−84	36	0.94	2.94*	0.32	1.44
17	Thalamus L (cTtha)	−12	−21	12	1.89	3.07*	1.94	1.27

*Note:* A10m = medial area 10, A45r = rostral area 45, ASTS = anterior temporal sulcus, A37mv = medioventral area 37, A37lv = lateroventral area 37, V5/MT+ = area V5/MT+.

Abbreviations: BNA, Brainnetome Atlas; BSR, bootstrap ratio; cTtha, caudal temporal thalamus; iOccG, inferior occipital gyrus; mOccG, middle occipital gyrus; msOccG, medial superior occipital gyrus; rLingG, rostral lingual gyrus, rpSTS, rostroparietal superior temporal sulcus; vmPOS, ventromedial parietooccipital sulcus.

**Table 4:** longitudinal effects of concussion on *c*_1_, averaged over all significant ROIs in Table 3. (left) longitudinal analysis using an LMM to compare postacute imaging sessions (SUB, RTS, 1MO, 1YR) to ACU. (Right) Cross‐sectional analysis using a GLM to compare concussed imaging sessions (ACU, SUB, RTS, 1MO, 1YR) to uninjured controls. Statistics include coefficients of effect *b*, 95% confidence intervals (95% CIs), BSRs, and *p*‐values. Significant longitudinal change relative to ACU is noted with “*” and significant cross‐sectional difference relative to controls is noted with “**” (FDR = 0.05 threshold)

	Longitudinal (LMM)				Cross sectional (GLM)			
	*b*	95%CI	BSR	*p*	*b*	95%CI	BSR	*p*
ACU	—	—	—	—	−0.030	−0.077, 0.016	−1.24	.206
SUB	0.038	−0.014, 0.102	1.43	.152	−0.006	−0.047, 0.037	−0.29	.782
RTS	0.118	0.077, 0.163	5.38	<.001*	0.083	0.037, 0.129	3.58	<.001**
1MO	0.079	0.024, 0.135	2.86	.008*	0.047	0.003, 0.090	2.06	.032
1YR	0.071	0.005, 0.137	2.03	.046	0.039	−0.011, 0.086	1.58	.124

Abbreviations: BNA, Brainnetome Atlas; BSR, bootstrap ratio; FDR, false discovery rate; GLM, general linear model; LMM, linear mixed model; ROI, region of interest.

### 3.4 Covariate effects

**Figure 4:**
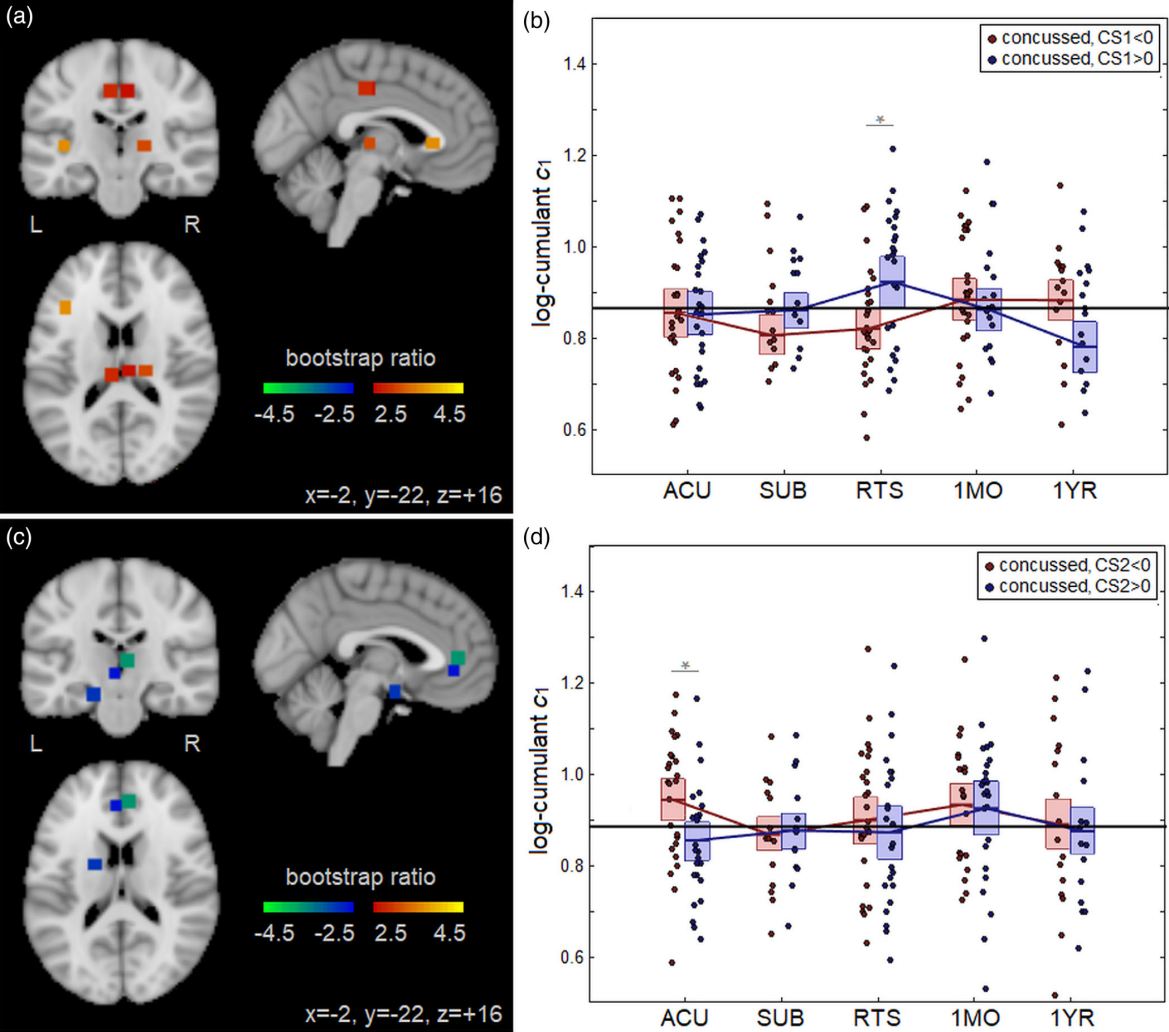
(a) Brain regions of interest (ROIs; square patches) where *c*_1_ during concussion recovery is significantly affected by CS1. (b) Distribution of concussed athlete *c*_1_ values, averaged over significant ROIs, plotted for each imaging session; distributions are plotted for CS1 < 0 and CS1 > 0 subgroups. (c) Brain ROIs (square patches) where *c*_1_ during concussion recovery is significantly affected by CS2. (d) Distribution of concussed athlete *c*_1_ values, averaged over significant ROIs, plotted for each imaging session; distributions are plotted for CS2 < 0 and CS2 > 0 subgroups. For the distribution plots, horizontal red/blue lines denote group means and boxes indicate 95% confidence intervals; distribution means are connected between sessions by solid red/blue lines; the mean *c*_1_ value for controls is plotted as the thick horizontal black line. The *c*_1_ values were obtained from ROIs showing significant interaction with CS1 or CS2, noted with “*” (false discovery rate [FDR] = 0.05 threshold)

The clinical CS1 (symptoms + days to RTP) was associated with significant effects on *c*_1_ during concussion recovery, with positive effects seen at RTS within the left inferior frontal gyrus (rostral area 45; CoM = −39, −6, 6; BSR = 3.61), left cingulate gyrus (caudal area 23; CoM = −6, −24, 42; BSR = 2.82), right cingulate gyrus (caudal area 23; CoM = 6, −21, 42; BSR = 2.62) and left thalamus (rostral temporal; CoM = 18, −21, 3; BSR = 3.07), depicted in Figure 4a. Averaging over significant brain regions, a positive effect was seen at RTS (*b* = 0.038; 95%CI: 0.022, 0.056; BSR = 4.43; *p* < .001), indicating that concussed athletes with greater CS1 scores had higher *c*_1_ values in these areas at RTS. Figure 4b plots the distribution of participant *c*_1_ values as a function of imaging session, averaged over significant ROIs. For athletes with CS1 < 0 (lower symptoms, fewer days to RTS) average *c*_1_ was below the average control value from ACU to RTS and slightly elevated from 1MO to 1YR, whereas for athletes with CS1 > 0 (higher symptoms, more days to RTS) average *c*_1_ was elevated at RTS, followed by a decline below control values by 1YR.

Significant effects of clinical CS2 (symptoms − days to RTP) on *c*_1_ were also identified, with negative effects seen at ACU in the left cingulate gyrus (subgenual area 32; CoM = −3, 39, −3; BSR = −2.64), right cingulate gyrus (subgenual area 32; CoM = 6, 42, 6; BSR = −3.67) and left amygdala (medial; CoM = −18, −3, −18; BSR = −2.91), as shown in Figure 4c. Averaging over significant brain regions, a negative effect was seen at ACU (*b* = −0.071; 95%CI: −0.106, −0.042; BSR = −4.76; *p* < .001), indicating that concussed athletes with greater CS2 scores had lower *c*_1_ values in these brain regions at early injury. Figure 4d plots the distribution of participant *c*_1_ values as a function of imaging session, averaged over significant ROIs. For athletes with CS2 < 0 (lower symptoms, more days to RTS) average *c*_1_ was above the average control value at ACU but declined toward control values in later imaging sessions, whereas for athletes with CS2 > 0 (higher symptoms, fewer days to RTS) average *c*_1_ was below control values at ACU but increased toward controls in later imaging sessions.

## 4 DISCUSSION

There is growing evidence that scaling analysis of functional brain dynamics captures important information about cognition and brain health. However, to date, most of the BOLD fMRI research in this domain has focused on healthy adults and has examined how scaling is modulated by cognitive tasks and other sources of effort (Barnes et al., 2009; Churchill et al., 2016; Ciuciu et al., 2012; He, 2011). The present study is the first to examine the relationship between scaling behavior in resting‐state fMRI and sport‐related concussion, with reference to a large control group. This study is also the first to show that functional brain dynamics are significantly altered in the course of concussion recovery, with effects that are modified by clinical variables of acute symptom severity and time to RTS.

The initial analyses of uninjured control athletes identified strong BOLD scaling behavior (*c*_1_) with significant multifractality (*c*_2_) throughout the healthy resting brain. Both log‐cumulant values had highest absolute values in cortical regions, particularly frontal, parietal and cingulate, whereas absolute values tended to be lowest in subcortical regions. This is consistent with an earlier monofractal scaling analysis of resting‐state fMRI (He, 2011), which reported high *H* values for nodes of the default mode and frontoparietal attention networks, along with low *H* values for noncortical network nodes. A previous multifractal scaling analysis of resting‐state fMRI (Ciuciu et al., 2012) also reported the highest scaling in networks encompassing dorsoparietal, visual, and parieto‐cingulate regions. Given the large control cohort in this study and the supporting literature, we may therefore conclude that BOLD scaling shows consistent spatial organization in the brain, with the highest values for cortical regions implicated in higher level processing, for example, executive function, attention, and interoception (Behrmann, Geng, & Shomstein, 2004; Critchley, Wiens, Rotshtein, Öhman, & Dolan, 2004; Fuster, 2000) and the lowest values in subcortical regions.

Further analyses of the controls identified significant demographic effects on scaling (*c*_1_) but not multifractality (*c*_2_). Greater age was associated with increased *c*_1_, albeit for a spatially limited set of ROIs, likely due to the narrow age band being studied. Nevertheless, results are consistent with a large‐scale study of resting electrophysiology and brain maturation (Smit et al., 2011), which reported increasing long‐range dependence with age until a plateau at 25–30 years, with similar trends in functional brain topology (Dosenbach et al., 2010). The observed effects were mainly localized in somatosensory and motor areas. One explanation is that athletes are improving skills involving these domains during their training, leading to increased neural efficiency (Guo, Li, & Yu, 2017; Wei & Luo, 2010) and therefore more scale‐free signal among older athletes. In addition, female athletes exhibited lower BOLD scaling than male athletes, within a distributed set of prefrontal, occipital, and subcortical regions. Previous studies have reported sex differences in functional brain topology (Tian, Wang, Yan, & He, 2011; Zuo et al., 2010); however, to our knowledge, sex differences in resting‐state BOLD scaling dynamics have not been previously examined. History of concussion had no significant effects on BOLD scaling, which aligns with nonsignificant findings in studies of resting‐state functional connectivity and task‐based activation for athletes with a history of concussion (Churchill, Hutchison, Leung, Graham, & Schweizer, 2017; Terry et al., 2012). Similarly, among concussed athletes, *c*_1_ in posterior brain regions did not show significant effects of age but did show effects of sex that were comparable to uninjured controls. In contrast with the controls, however, concussed athletes with a history of concussion had significantly reduced posterior *c*_1_, suggesting a cumulative effect of repeated brain injury on BOLD dynamics during concussion recovery. This is consistent with prior literature, in which a history of concussion was associated with greater disturbances in functional connectivity and neurometabolites during recovery from subsequent sport‐related concussion (Johnson et al., 2012; Vagnozzi et al., 2008).

The analyses of concussed athletes identified significant longitudinal changes in BOLD scaling (*c*_1_), whereas multifractality (*c*_2_) showed no evidence of longitudinal change. These findings partly support our first study hypothesis, as BOLD scaling values were lowest at acute injury and increased in later imaging sessions. The findings are also congruent with prior literature, in which reduced BOLD scaling is associated with greater cognitive burden (Barnes et al., 2009; Churchill et al., 2016; Ciuciu et al., 2012; He, 2011) and mental distress (Churchill et al., 2015; Tolkunov et al., 2010). Unlike conventional BOLD measures of task‐related activity and functional connectivity, which may show either increases or decreases, scale‐free dynamics consistently show suppression associated with these cognitive and psychological stressors. This has been observed as a common feature of many biological systems, where a reduction in scaling behavior signals a loss of system complexity (Goldberger et al., 2002) and thus a more constrained, less adaptive system. The present study findings suggest that acute concussion, which is associated with impairments across multiple domains, including cognition (Echemendia, Putukian, Mackin, Julian, & Shoss, 2001) and emotion regulation (McCrory et al., 2017), may similarly involve disruptions in the scale‐free dynamics of underlying brain function. However, our second study hypothesis was not supported, for although the *c*_1_ values of concussed athletes were low relative to controls, the difference was nonsignificant. This is consistent with previous literature which has reported heterogeneous effects of concussion on resting brain function in the first week post‐injury (Churchill et al., 2017a). These findings also reinforce that the effects of concussion on brain function are modest, compared to more severe forms of TBI (McAllister, Flashman, McDonald, & Saykin, 2006).

Intriguingly, post‐RTS findings for concussed athletes differ from those predicted by our first study hypothesis. Instead of a continual increase in *c*_1_ over time, the values for concussed athletes peaked at RTS, followed by a decline up to 1 year post‐RTS. The *c*_1_ values of concussed athletes at RTS were also significantly elevated compared to uninjured controls. These findings suggest that persistent concussion‐related disturbances in brain function are present at RTS, which is consistent with prior studies which reported elevated resting‐state functional connectivity and task‐based activation after RTS (Churchill, et al., 2017b; Lovell et al., 2007). The results are surprising, as elevated scaling is thought to indicate a less taxed, more adaptive brain (Barnes et al., 2009; Churchill et al., 2016). The elevated *c*_1_ values may therefore reflect adaptive changes that serve to maintain brain function postconcussion, similar to functional hyperconnectivity often reported after TBI (Hillary & Grafman, 2017). Alternatively, these elevations may reflect an improved disposition relative to “normal” controls, as the athletes had recently been declared fit for unrestricted sport participation. This is supported by the athletes having significantly lower symptom scores at RTS compared to their preseason baseline. Such an effect was also observed in a previous study, where recently cleared athletes scored higher on multiple psychological measures relative to matched athletic controls (Hutchison et al., 2017). Another potential contributor may be interruption in sport practice, that is, increased physiological rest. We believe it unlikely to be the primary driver of the observed effects, however. Despite substantial variability in time to RTS, the brain regions with significantly elevated *c*_1_ at RTS did not show significant effects of CS, which is highly correlated with recovery time; nevertheless, further work is needed to validate these conclusions. Irrespective of the underlying mechanism, concussion‐related abnormalities seen at RTS gradually dissipated over subsequent sessions, indicating that concussion‐related disturbances in BOLD dynamics had largely dissipated by 1 year post‐RTS.

The effects of concussion on BOLD fMRI scaling values were mainly observed in posterior brain regions implicated in visual function and processing, with less frequently identified effects in temporal and thalamic regions. This has significant implications, particularly given the frequent reporting disturbances in visual function following concussion and mild TBI (Brahm et al., 2009; Capó‐Aponte, Urosevich, Temme, Tarbett, & Sanghera, 2012). However, an alternative explanation for the spatial distribution of effects is that the dense vascularization and relative functional homogeneity within these brain regions may better facilitate the accurate estimation of subtle changes in scaling behavior for BOLD signals. The localization of concussion effects in this study are nevertheless consistent with a meta‐analysis conducted by (Eierud et al., 2014), which showed that mild TBI is associated with enhanced posterior functional brain connectivity, supporting a distinct pattern of functional response in these brain regions after a concussion.

The analysis of CS1 and CS2 supported our third study hypothesis, as the course of brain recovery for *c*_1_ showed significant effects of symptom severity and time to RTS. Based on the analysis of CS1, athletes with greater acute symptom burden and prolonged recovery (CS1 > 0) had elevated *c*_1_ at RTS followed by a decline 1 year later. As the differences in brain response in this subgroup are observed at medical clearance, it remains unclear whether they represent greater adaptive neural response to injury, greater elevation in mood at RTS due to the protracted nature of recovery, or a consequence of greater physiological rest. The significant brain regions, which include inferior frontal, anterior midcingulate and rostral thalamic ROIs, are consistently implicated in motor control (Hoffstaedter et al., 2014; Lissek et al., 2007; Peyron et al., 2007). These findings suggest that motor function may be a key aspect of recovery in athletes with greater overall severity of clinical outcomes. This hypothesis is supported by a previous study of concussed athletes, which found that athletes with longer time to RTS had significantly elevated functional connectivity of motor networks at RTS (Churchill, et al., 2017b). Nonetheless, given the spatial sparsity of results, further research is needed to replicate and validate these study findings.

For the analysis of CS2, athletes with a greater acute symptom burden but relatively fast recovery (CS2 > 0) tended to have reduced *c*_1_ at acute injury, while those with lower symptoms but a relatively slow recovery (CS2 < 0) had the opposite response. In both cases, the effects were only present at acute injury and had normalized to near‐control values at the time of subacute injury. The significant brain regions, which included subgenual anterior cingulate and amygdala ROIs, are both involved in emotion regulation (Etkin, Egner, & Kalisch, 2011; Haas, Omura, Constable, & Canli, 2007; LeDoux, 2003). Hence, acute disturbances in BOLD scaling for brain regions involved in emotion regulation may be an early indicator of atypical recovery time, relative to what is anticipated from symptom assessments. A possible interpretation is that athletes with CS2 > 0 have high initial worry about postconcussion outcomes, with a tendency toward greater overall symptom reporting, while those with CS2 < 0 have less worry and tend to underreport symptoms. This hypothesis is supported by prior literature showing reduced BOLD scaling in individuals with greater self‐reported worry and anxiety (Churchill et al., 2015). Given the complex interplay between concussion pathophysiology, anxiety, and emotion (Mainwaring, Hutchison, Camper, & Richards, 2012; Sandel, Reynolds, Cohen, Gillie, & Kontos, 2017), future work should combine functional neuroimaging with more detailed assessments of emotional state from acute injury to RTS, in order to validate these study findings.

In terms of clinical utility, our understanding of BOLD dynamics and their relationship with concussion as a clinical syndrome remain in its early stages. Nevertheless, this study provides new insights into cerebral pathophysiology, and the relationship between brain recovery and clinical determinants of RTS. As previously noted, this study provides further convergent evidence of incomplete recovery of brain function at RTS (Churchill, et al., 2017b; Lovell et al., 2007), which may be relevant in the refinement of concussion management guidelines. This is also a promising tool for future investigation of sport‐related concussion, as analyses can be conducted on resting‐state data and do not require the selection of seed regions and/or networks of interest, as in more ubiquitous measures of functional connectivity. Even more promising, scale‐free brain dynamics are, by definition, conserved across a range of timescales and potentially across different measures of brain function, as scale‐free electrophysiological fluctuations are thought to underlie scale‐free BOLD dynamics (Van de Ville et al., 2010). This suggests that the present findings may be readily translated into lower‐cost and more portable devices, such as functional near‐infrared spectroscopy (fNIRS) and electroencephalography (EEG), which are more easily implemented for patient assessment in a clinical setting.

This study provided evidence that the temporal dynamics of spontaneous BOLD fluctuations evolve over the course of concussion recovery. Nevertheless, study findings should be interpreted in the context of its limitations. There was some attrition of concussed athletes, which may lead to biased parameter estimates. However, as noted in Section 2.1, drop out was not significantly dependent on demographics (age, sex, and concussion history) or clinical factors (symptom severity, time to RTS). Moreover, to mitigate bias and improve estimation efficiency, longitudinal analyses used LMMs and cross‐sectional analyses used multiple imputation techniques. Further work is also needed to determine whether the present study findings generalize beyond university‐level athletes. We identified significant demographic effects on BOLD scaling within a relatively homogeneous athlete cohort, highlighting the importance of evaluating scaling behavior across a broader range of ages and other sport and nonathlete cohorts. In addition, although history of concussion was incorporated into analyses of both control and concussed athletes, this was based on self‐reported history, which may be subject to errors and/or reporting bias. Future work should include more comprehensive documentation of concussion history and long‐term follow‐up to better understand the cumulative effects of multiple concussions on BOLD scaling.

In conclusion, these findings present the first examination of concussion recovery and BOLD fMRI multifractal scaling behavior, showing longitudinal effects related to recovery predominantly in brain regions involved in visual function and processing. These effects were most extensive at RTS, providing further evidence of ongoing functional brain changes beyond medical clearance. Future research should examine other measures of brain physiology in the concussion context, such as fNIRS and EEG, thereby assessing scaling of brain activity over different timescales. In addition, the effects of concussion on scaling should also be examined during cognitive tasks, to better understand how concussion affects the brain's ability to regulate mental effort. Overall, these results underscore the promise of such analysis techniques in future research and suggest that they should complement standard connectivity‐based analyses of resting‐state fMRI data.

## Supporting information


**Table S1** athlete numbers by sport, for both male (M) and female (F) groups, for N = 167 controls and N = 61 concussed athletes.

## Data Availability

The data that support the findings of this study are available from the corresponding author upon reasonable request.
