# Supplementary material for: Scale‐free functional brain dynamics during recovery from sport‐related concussion
Source: Hum Brain Mapp. 2020 Apr 29;41(10):2567–82. doi: 10.1002/hbm.24962 (PMC7294069; doi:10.1002/hbm.24962)
Supplement: Supplementary file 1 — Table S1 athlete numbers by sport, for both male (M) and female (F) groups, for N = 167 controls and N = 61 concussed athletes. [file HBM-41-2567-s001.docx]

**Table S1:** athlete numbers by sport, for both male (M) and female (F) groups, for N=187 controls and N=61 concussed athletes.

| **CONTROL** | **CONCUSSION** |
| --- | --- |
| Squash (1M)  Water polo (1M)  Lacrosse (10M* / 6F)  Basketball (3M / 11F)  Rugby (6M* / 11F*)  Football (11M*)  Soccer (17M / 11F)  Hockey (27M* / 36F)  Volleyball (14M / 22F) | Mountain biking (1F)  Water polo (1M / 1F)  Lacrosse (3M* / 3F)  Basketball (2M / 2F)  Rugby (7M* / 14F*)  Football (7M*)  Soccer (1F)  Hockey (7M* / 6F)  Volleyball (3M / 3F) |

* collision sports, defined as involving routine, purposeful body-to-body contact^47^
